# Increasing Fracture Risk Associates With Plasma Circulating MicroRNAs in Aging People’s Sarcopenia

**DOI:** 10.3389/fphys.2021.678610

**Published:** 2021-06-07

**Authors:** Nana He, Yuelin Zhang, Yue Zhang, Beili Feng, Zaixing Zheng, Dongjuan Wang, Shun Zhang, Honghua Ye

**Affiliations:** ^1^Department of Experimental Medical Science, HwaMei Hospital, University of Chinese Academy of Sciences, Ningbo, China; ^2^Key Laboratory of Diagnosis and Treatment of Digestive System Tumors of Zhejiang Province, Ningbo, China; ^3^Department of Cardiology, HwaMei Hospital (Previously Named Ningbo No. 2 Hospital), University of Chinese Academy of Sciences, Ningbo, China

**Keywords:** sarcopenia, circulating microRNAs, fracture, aging, plasma

## Abstract

Aging generally coincides with a gradual decline in mass and strength of muscles and bone mineral density (BMD). Sarcopenia is closely linked to osteoporosis in the elderly, which can lead to abnormal gait, balance disorders, and dysfunctions, as well as increase in the risks of falls, fractures, weakness, and death. MicroRNAs (miRNAs, miRs) are a kind of short and non-coding RNA molecules but can regulate posttranscriptional protein expression. However, we have known little about their participation in age-associated osteoporosis and sarcopenia. The current study aims to confirm those miRNAs as biomarkers for age-related reduction in muscular atrophy associated with human blood fractures. In our study, 10 fracture-risk-related miRNAs (miR-637, miR-148a-3p, miR-125b-5p, miR-124-3p, miR-122-5p, miR-100-5p, miR-93-5p, miR-21-5p, miR-23a-3p, and miR-24-3p) were analyzed. For the initial screening, we determined the abundance of fracture-risk-associated miRNAs by RT-PCR most frequently detected in enrolled 93 elderly with sarcopenia and non-sarcopenia, respectively. Statistically, the relative expression levels of plasma miR-23a-3p, miR-93-5p, and miR-637 in the sarcopenia group were significantly lower than that in the non-sarcopenia group, while the levels of other miRNAs did not change significantly. Moreover, we showed that the levels of ASM/height^2^, handgrip strength, and 4-m velocity in the sarcopenia group were significantly lower than in the non-sarcopenia group. Whereafter, we expanded the sample for further detection and analysis and revealed that the levels of plasma miR-23a-3p, miR-93-5p, and miR-637 in the sarcopenia group were significantly lower than that in the non-sarcopenia group, which is consistent with the initial screening experiment. From our analysis, changes in levels of plasma miR-93-5p and miR-637 were dramatically related to ASM/height^2^. Furthermore, changes in miR-23a and miR-93-5p were significantly affected by ASM/height^2^ in female individuals, with no significant correlations between miRNAs changes and these diagnostic indexes in male individuals after adjusting sex. The study showed that plasma miRNAs changed in an aging-related sarcopenia manner and were associated with increased fracture risk. In aging patients, plasma miR-23a-3p, miR-93-5p, and miR-637 have the potential as biomarkers of sarcopenia, which can affect the development of physiological dysfunction and may be also used in the fracture risk assessment of these patients.

## Introduction

Sarcopenia is a common geriatric syndrome (Marzetti et al., 2017; Larsson et al., 2019; Tournadre et al., 2019). In normal aging, a gradual decline occurs after a maximum of about 30 years of age and a decline of 20–40% annually after 40 years of age, leading to corresponding muscle dysfunction, which also can be considered as one of the indicators of frailty in the elderly. Currently, sarcopenia is defined as a clinical syndrome related to aging-related loss such as skeletal muscle mass, strength, and function, which will cause an increase in the risk of occurring physical disability, poor quality of life, and mortality (Landi et al., 2018). Sarcopenia has widespread seriousness of harm, which will lead to abnormal gait, balance disorders, and incapacitation, with an increased risk of falls, fractures, weakness, and death in the elderly (Yang et al., 2019). It also can be regarded as a factor of risk for poor prognosis of some chronic diseases such as cirrhosis, type 2 diabetes, and tumor (Angulo et al., 2016). Therefore, effective measures should be taken to slow down or even reverse the progression of sarcopenia in the elderly to the occurrence of adverse clinical outcomes, with the improvement in patients’ quality of life. Exercise is positive to the muscle strength and body function in the elderly. Nutritional supplementation can improve the exercise effect of healthy people and can improve the effect of an exercise intervention on sarcopenia. Given the positive role of resistance exercise on human muscle mass, more and more studies have added resistance exercise to the treatment of sarcopenia. A study showed that one cycle (12–16 weeks) of resistance training increased the subjects’ thigh circumference by 11.4% and muscle volume by 3.8% (Van Roie et al., 2013). What is more exciting, plenty of studies have proved that muscle growth caused by resistance exercise can occur at any age, even elderly people in their 1990s (Hunter et al., 2000; Westcott, 2009). According to Khadijeh et al., exercise in water can improve muscular balance and muscle strength and provide appropriate postural mobility without the fear of falling in older adults with inappropriate body shapes (Irandoust et al., 2018). Studies have shown that regular physical activity can reduce abdominal fat intake, improve musculoskeletal control, reduce lower back pain, and improve the quality of balance and walking speed in the elderly (Irandoust et al., 2018, 2019). Similarly, pilates exercise can decrease body fat mass and improve muscle atrophy, balance, and walking speed in inactive middle-aged women (Seghatoleslami et al., 2018). It was shown that skeletal muscle atrophy and skeletal muscle strength were significantly improved in elderly patients with sarcopenia after adequate vitamin D and amino acid treatment (Bauer et al., 2015). It is now thought that nutrition combined with exercise can better improve muscle strength and function. Studies have found that combining resistance exercise with protein and vitamin D supplements is the most effective way to improve sarcopenia or myasthenia in the elderly (Anton et al., 2018; Yamada et al., 2019; Eshaghi et al., 2020). However, the complex pathogenesis of sarcopenia and numerous influencing factors have not been fully understood.

As the society gets aging, the prevalence of musculoskeletal diseases is growing rapidly, showing that bone is closely related to muscle tissue: both of them are not only adjacent to the anatomical location but also have common paracrine and endocrine regulation, similar molecular signal regulation pathways, and common therapeutic targets and drugs (Bonewald et al., 2013; Girgis, 2015), which are biologically and functionally in line with increasing the risks of fracture in the elderly (Wong et al., 2019). Aging is involved in the loss of bone and muscle functions (Laurent et al., 2019). In particular, losing mass, strength, and function of muscles allied to aging significantly increases the risks in causing osteoporosis and fractures; furthermore, age-related decline in bone strength will also significantly increase the incidence of sarcopenia (Edwards et al., 2015; Oliveira and Vaz, 2015). The increased risk in causing fracture in patients with sarcopenia and osteoporosis is due to decreased muscle mass and strength, decreased bone density, and limited movement (Tarantino et al., 2016; Steihaug et al., 2017). Elderly people with sarcopenia are three times more likely to fall, and there is growing evidence that sarcopenia is closely linked to fractures (Marques and Queirós, 2018; Yeung et al., 2019). On top of that, there have been recent reports of a high prevalence of sarcopenia in patients with fractures, which is alarming for clinicians. However, there are currently still few clinical data to suggest that there is a causal relation between osteoporosis and sarcopenia.

MicroRNAs (miRNAs) are endogenous, non-coding RNAs that are 19–25 nucleotides long, which are able to achieve negative regulation of gene expressing at the posttranscription level through degradation or translational inhibition of messenger RNA (mRNA) (Saliminejad et al., 2019). A single miRNA, as a central regulator of gene networks, can directly or indirectly regulate the expression of hundreds of gene targets, which may be regulated by a suite of miRNAs as well. miRNAs are critical in various physiological and pathological processes including sarcopenia with its deregulation (Soriano-Arroquia et al., 2016; Yin et al., 2020). Interestingly, miRNAs are very stable and can be easily detected in blood circulation (e.g., plasma), where the profile of miRNAs can reflect health status, suggesting that circulating miRNAs (c-miRNAs) are considered as new, potential, and even more sensitive biomarkers for disease diagnosis and related treatment (Kosaka et al., 2010; Zendjabil et al., 2017; Gareev et al., 2020), whose signature for patients with osteoporotic fractures has been reported. The level of one of these osteoporosis-associated miR-21-5p and other nine related ones has been predicted to be fracture risk in patients with osteoporosis more recently (Seeliger et al., 2014; Panach et al., 2015; Kocijan et al., 2016; Kelch et al., 2017). It has recently been reported that muscles and bones are regulated by many common genes, endocrine regulatory networks, and signaling pathways. These common molecular regulations can significantly increase the incidence of sarcopenia and osteoporosis in the elderly, which often coexist (Chen et al., 2019; Reiss et al., 2019). Considering that bone and muscle are closely related to mechanotransduction and metabolic signaling, we can reasonably assume that sarcopenia can be assessed by measuring bone-specific c-miRNAs which can reflect resorption and formation of bones. Nevertheless, the regulation of c-miRNA in osteoporosis, fractures, tendonopenia, and a variety of other human conditions has not been totally understood yet.

Finally, considering the link between bone and muscle in mechanotransduction and metabolic signaling, osteoporosis and sarcopenia are both key factors in fractures, which may be useful to contain sarcopenia status in fracture risk model. Thus, this study is mainly to explore the relationship between well-functioning specific circulating miRNAs and reduced muscular atrophy and fracture risk in the elderly, and second, it is to determine the associations between these specific c-miRNAs and musculoskeletal variables. Specifically, we identified changes in circulating miRNA levels in the elderly with and without nodular reduction. More longitudinal studies are needed to assess whether circulating miR-23a-3p, miR-93-5p, and miR-637 have the potential as biomarkers of sarcopenia in the elderly.

## Materials and Methods

### Study Population

In the present study, participants were chosen from Ximen Community of Ningbo, China. There were 1,047 elderly people aged 65 years old or more who were involved in our examination and completed a comprehensive geriatric assessment from November 2016 to March 2017. Inclusion criteria were the following: people aged 65 years old or more who were eligible to participate and people who can independently finish a comprehensive geriatric assessment, including tests of walking speed, grip strength and muscle mass. Exclusion criteria were the following: people refusing to take part in this study, people failing to complete the items that they were required to be inspected independently, and people who were aged <65 years old. Based on the above criteria, participants with the record of hand strength, 4-m speed or body composition (*n* = 28), or measurement of baPWV (*n* = 13) or the filled questionnaire (*n* = 4) were excluded. Thus, a total of 1,002 participants were analyzed. According to the diagnostic criteria of the Asian Working Group for Sarcopenia (AWGs) for sarcopenia, the participants were further divided into sarcopenia group (*n* = 93) and non-sarcopenia group (*n* = 93), as the matching factors of gender and age.

The study was carried out in accordance with the principles of the Declaration of Helsinki. All participants were informed in advance of the procedures, benefits, and probable adverse events associated with the protocol. Study subjects provided written informed consent, including permission to use the collected data for research purposes only. The study protocol has been approved by the Ethics Committee of Ningbo No. 2 Hospital.

### Definition of Sarcopenia

Sarcopenia was diagnosed as low muscle mass, low muscle strength, and/or low physical performance by the Asian Working Group for Sarcopenia (AWGS) criteria. For low muscle mass, ASM/Ht^2^ was <7.0 kg/m^2^ for male and 5.7 kg/m^2^ for female individuals, respectively. For low muscle strength, male handgrip strength was <2 kg, and female handgrip strength was <18 kg, respectively. Four-meter walking velocity <0.8 m/s could low physical performance.

### Assessment of Muscle Strength and Physical Performance

The direct segmental multifrequency bioelectrical impedance analysis was used to analyze body composition features. Appendicular skeletal muscle mass (ASM) was summed as the total skeletal muscle in arms and legs. Relative skeletal muscle mass index (ASM/Ht^2^) was defined as ASM divided by height squared in meters. Muscle strength was collected to the nearest 0.1 kg with an accurate handgrip dynamometer. Four-meter walking speed was tested on a straight corridor with a 6-m mark on the ground.

### Other Measurements

Each participant was asked about age, sex, occupation, medical history, drug intake, smoking, and drinking habits via standardized questionnaires by experienced staff. Height and waist circumference were measured to the nearest 0.5 cm. Body mass index was weight in kilograms divided by the height squared in meters. Office blood pressure was measured with the Omron HEM-1300 monitor (Omron Healthcare, Inc., Kyoto, Japan). After resting in the sitting position for at least 5 min, the subjects took three blood pressure readings in a row, as recommended by the European Society of Hypertension. In the analysis, the mean office hypertension of three readings was blood pressure (BP) of at least 140 mmHg systolic or 90 mmHg diastolic.

In order to evaluate bone mineral density (BMD), all the participants underwent calcaneal ultrasound osteometry, with the analysis of BMD according to the manufacturer’s recommendations. Results were expressed as *T*-scores. *T*-value was obtained by subtracting the measured value from the peak value of normal young people’s bones. The smaller the difference, the better the bone quality. This value is used to diagnose osteoporosis and predict fracture risk.

Each participant was assessed for physical activity function through the International Physical Activity Questionnaire (IPAQ), which was designed specifically to assess physical activity in epidemiological studies of people over 65 years old.

Venous blood samples were obtained after overnight fasting, for the automatic enzymatic analysis on serum total cholesterol, high density lipoprotein cholesterol, serum creatinine, uric acid, and plasma glucose. Hypertension was caused by a previous diagnosis or the use of antihypertensive drugs. Diabetes mellitus was defined as having a glycosylated hemoglobin level of 7.0% or higher, taking antidiabetic medications, or having a history of diabetes. Dyslipidemia was defined as having a total cholesterol concentration higher than 5.0 mmol/L, with high-density lipoprotein (HDL) below 1.2 mmol/L in women or 1.0 mmol/L in men, or having been on lipid-lowering medications.

### International Physical Activity Questionnaire-Short Forms

The short IPAQ administered by the interviewers determined the frequency and duration of moderate and vigorous leisure, transportation, and occupational physical activity, walking physical activity, and inactivity during the past week. IPAQ has created three physical activity categories, namely, walking, moderate intensity, and high intensity.

Participants are asked about the frequency of different intensity activities in 1 week and the accumulated time of each day. Each question was set up with examples of moderate, vigorous, and walking activity for the participants to choose from, along with physiological cues for breathing and heart rate to help them recall activity at the appropriate intensity. One metabolic equivalent task (MET)-minute is defined as the intensity of a MET multiplied by the minutes of activity weekly. A MET roughly equals to active metabolic rate divided by resting metabolic rate, which represents the energy expended while sitting quietly at rest (21). MET intensity levels used for the iPAQ score are vigorous (8.0 METs), moderate (4.0 METs), and walking (3.3 METs). The principle of data outlier elimination is as follows: First, the daily cumulative time of each activity needs to be converted into minutes. Any missing data on activity frequency or time will not be included in the analysis. Assuming that each person had at least 8 h of sleep per day, if the cumulative time of three kinds of physical activity reported by the individual exceeded 960 min (16 h) per day, the individual was not included in the analysis.

### Plasma Sampling and RNA Isolation

To obtain plasma, in silicone-coated serum tubes with increased silica act clot activator, venous blood was collected and then treated within 1 h after collection. The blood samples were separated by centrifuge at 845 × *g*, at 4°C for 15 min, with plasma and erythrocytes separated. Plasma were collected in aliquots into RNase/DNase-free tubes and stored at −80°C for further analysis.

Total RNA was isolated from the plasma with a mirVana PARIS isolation kit (Ambion, Austin, Texas) under the manufacturer’s instructions. To avoid discrepancies in results, all samples were extracted and analyzed in a single batch to minimize repeated freeze–thaw cycles of plasma samples. Briefly, total RNA was extracted from 400 μl of plasma. After adding the same volume of denaturing solution, the plasma miRNA level was normalized by adding 50 pmol/L of *Caenorhabditis elegans* miR-39 (cel-miR-39) as the peak control. All the samples were eluted with 100 μl of RNAse-free water.

### Analysis of Circulating miRNAs

The iScript complementary DNA (cDNA) reverse transcription kit (Bio-Rad) was used to reverse transcribe into cDNA from RNA. miRNA expressions were detected by Bulge-Loop TM miRNA qPCR Primer Sets (RiboBio) and quantitative reverse transcription polymerase chain reactions (qRT-PCRs) with iTaq^TM^ Universal SYBR Green Supermix (BIO-RAD) to quantify circulating miRNA levels. An Applied Biosystems 7900HT Fast Real-Time PCR device was used to perform all qRT-PCR reactions in triplicate. The patients with Ct values <35 were accepted for the analysis. The amplification efficiency of reference and studied miRNAs is near 100%, and the difference between the reference and studied miRNAs are <5%. Each sample was normalized to a reference sample across the plates. ΔCt values equals ΔCt = mean Ct_*miR*__–__*X*_ − mean Ct_*miR(reference)*_. The formula 2^(–Δ^ ^Δ^ ^*Ct)*^ was used to calculate the fold-change of RNA species with Cel-miR-39 as spike-in control.

### Statistical Analysis

GraphPad Prism 6 software (La Jolla, CA, United States) was used to performed statistical analysis. Means ± standard deviation (SD) represents subject characteristics, biochemical measurements, and general echocardiographic indexes. Through the analysis on qRT-PCR data, the 2^–Δ^ ^Δ^ ^*Ct*^ method was used to calculate the relative expression level for each miRNA, and data were as mean ± SD. An appropriate *t*-test was performed on the unpaired samples to analyze the miRNA changes. The Pearson’s method was used to conducted the correlation analysis between changes in circulating miRNAs and diagnostic indexes of sarcopenia (ASM/height^2^, handgrip strength and 4-m velocity) as appropriate for data distribution. *P* < 0.05 showed the statistical significance.

## Results

### Participant Characteristics

The cohort used in this study was in accordance with previously reported (Zhang et al., 2019). The characteristics of the participants in the different groups are shown in Table 1. The height was higher in the no-sarcopenia group than in the sarcopenia group (159.73 ± 0.78 vs. 156.03 ± 0.74, *p* < 0.05). Body mass was greater in non-sarcopenia group than that in sarcopenia group (64.72 ± 1.19 vs. 52.97 ± 0.80, *p* < 0.05). The mean BMI was statistically significantly higher in the non-sarcopenia group than that in the sarcopenia group (25.32 ± 0.41 vs. 21.75 ± 0.29, *p* < 0.05). These data confirmed that the underweight condition of sarcopenia is frequent. Both groups had similar levels of diabetes mellitus. The incidence of hypertension was significantly higher in the non-sarcopenia group than that in the sarcopenia group. The detailed anthropometric indexes of these participants are indicated in Table 2. ASM/height^2^, handgrip strength, knee extension, and 4-m velocity were significantly lower in the sarcopenia group than those in the non-sarcopenia group (*p* < 0.05). BMD, osteoarthritis, and physical activity scale were used for participants’ fracture evaluation. As expected, patients with sarcopenia had a higher incidence of reduced BMD, reduced daily physical activity index, and osteoarthritis, indicating a risk of fracture, compared with those without osteopenia (Table 3).

**TABLE 1:** Clinical characteristic of participants.

**Clinical parameters**	**Non-sarcopenia (*n* = 93)**	**Sarcopenia (*n* = 93)**	***P***
Age (years)	76.19 ± 0.58	76.15 ± 0.58	>0.05
**Gender (male/female)**			
Male	34 (36.6%)	34 (36.6%)	
Female	59 (63.4%)	59 (63.4%)	
Height (cm)	159.73 ± 0.78	156.03 ± 0.74	<0.05
Body mass (kg)	64.72 ± 1.19	52.97 ± 0.80	<0.05
BMI (kg/m^2^)	25.32 ± 0.41	21.75 ± 0.29	<0.05
Hypertension	83 (89.2%)	72 (77.4%)	<0.05
Diabetes mellitus	38 (40.8%)	26 (27.9%)	>0.05

**TABLE 2:** Anthropometric indexes of participants by sarcopenia status.

**Anthropometric indexes**	**Non-sarcopenia group**	**Sarcopenia group**	***p***
ASM/Height^2^ (kg/m^2^)	6.71 ± 0.10	5.63 ± 0.07	<0.05
Handgrip strength (kg)	24.18 ± 0.94	17.33 ± 0.55	<0.05
4-m velocit y (m/s)	1.12 ± 0.02	1.00 ± 0.03	<0.05
Knee extension (kg)	19.93 ± 0.81	14.76 ± 0.59	<0.05

**TABLE 3:** Evaluated parameters in participants with fracture.

**Physiological functions**	**Non-sarcopenia (*n* = 93)**	**Sarcopenia (*n* = 93)**	***P***
Bone mineral density (*T*-score)	−1.37 ± 0.11	−1.60 ± 0.12	>0.05
Osteoarthritis	9 (9.7%)	10 (10.8%)	>0.05
Physical activity (IPAQ)	1,376.29 ± 172.45	1,188.61 ± 85.73	>0.05

### Decrease in Fracture Risk-Associated Circulating MiR-23a-3p, MiR-93-5p, and MiR-637 in Response to Sarcopenia in the Elderly

**FIGURE 1:**
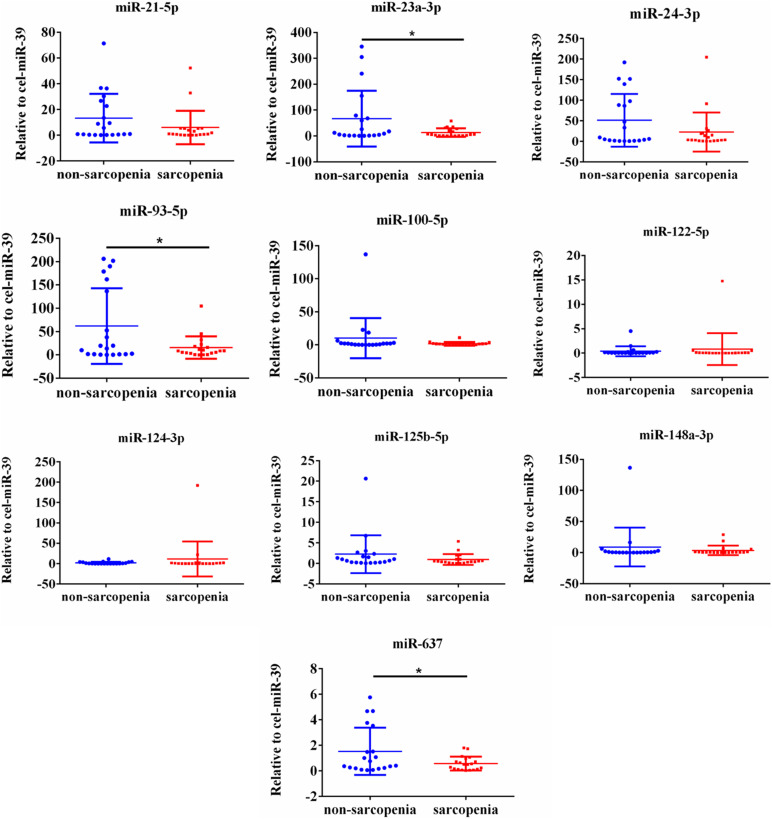
Changes in circulating microRNAs in response to sarcopenia in the elderly. The expression level of microRNAs (miRNAs) were normalized using spike-in cel-miR-39. Unpaired *t*-test was used for these data. *Compared to non-sarcopenia of the elderly; *P < 0.05; *n* = 20.

**FIGURE 2:**
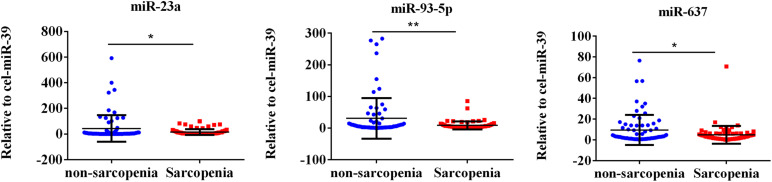
Fracture risk-associated circulating miR-23a-3p, miR-93-5p, and miR-637 decrease in response to sarcopenia in the elderly. The expression level of microRNAs (miRNAs) were normalized using spike-in cel-miR-39. Unpaired *t*-test was used for these data. *Compared to non-sarcopenia of the elderly; *P < 0.05, **P < 0.01; *n* = 73.

The expression of fracture risk-associated miRNAs [miR-21-5p (Seeliger et al., 2014), miR-23a-3p (Seeliger et al., 2014; Perksanusak et al., 2018), miR-24-3p (Kelch et al., 2017; Verdelli et al., 2020), miR-93-5p (Seeliger et al., 2014; Kelch et al., 2017), miR-100-5p (Ding et al., 2019), miR-122-5p, miR-124-3p (Qadir et al., 2015; Tang et al., 2017; Zou et al., 2017), miR-125b-5p (Lisse et al., 2013), miR-148a-3p (Hong et al., 2013; Verdelli et al., 2020), and miR-637 (Zhang et al., 2011; Gámez et al., 2014)] were determined. For the initial screening, we determined the expression level of miR-21-5p, miR-23a-3p, miR-24-3p, miR-93-5p, miR-100-5p, miR-122-5p, miR-124-3p, miR-125b-5p, miR-148a-3p, and miR-637 by RT-PCR most frequently detected in enrolled 93 sarcopenia elderly and 93 non-sarcopenia elderly plasma. The findings indicated that the levels of plasma miR-23a-3p, miR-93-5p, and miR-637 significantly decreased in the sarcopenia group compared to those in the non-sarcopenia group. On the contrary, miR-21-5p, miR-24-3p, miR-100-5p, miR-122-5p, miR-124-3p, miR-125b-5p, and miR-148a-3p did not change dramatically (Figure 1). According to continuous analyses, the experimental sample size was further expanded to verify the expression trend of these miRNAs that were differentially expressed, showing that the levels of miR-23a-3p, miR-93-5p, and miR-637 significantly decreased in the sarcopenia group compared to that in the non-sarcopenia group (Figure 2). The insulin signal decreases with age and is a special problem in patients with diabetes mellitus, which may be related to the c-miRNA response. We have excluded patients with diabetes mellitus from the analyzed data, suggesting that the levels of plasma miR-23a-3p, miR-93-5p, and miR-637 significantly decreased in the sarcopenia group compared to that in the non-sarcopenia group, with the same profile as the ones with diabetes mellitus (Supplementary Figure 1).

### Changes in Fracture Risk-Associated MiR-23a-3p, MiR-93-5p, and MiR-637 Correlate With ASM/height^2^, Handgrip Strength, or 4-M Velocity

**FIGURE 3:**
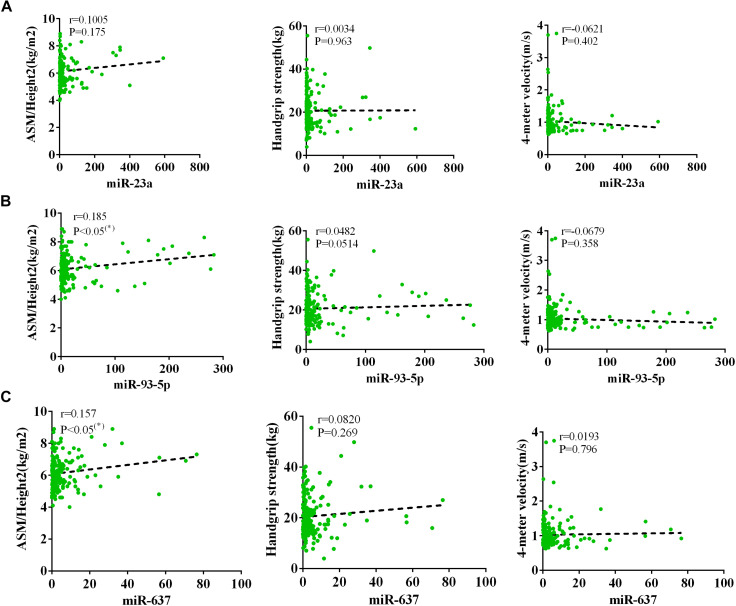
Correlation analysis between the changes in **(A)** miR-23a-3p, **(B)** miR-93-5p, and **(C)** miR-637 and ASM/height^2^ (kg/m^2^), handgrip strength (kg), and 4-m velocity (m/s). The correlation between the changes in miR-23a-3p, miR-93-5p, and miR-637 and the diagnostic indicators of sarcopenia (ASM/height^2^, handgrip strength, and 4-m velocity) in sarcopenic and non-sarcopenic subjects. Pearson’s method was used for correlation analyses;*P < 0.05; *n* = 186.

**FIGURE 4:**
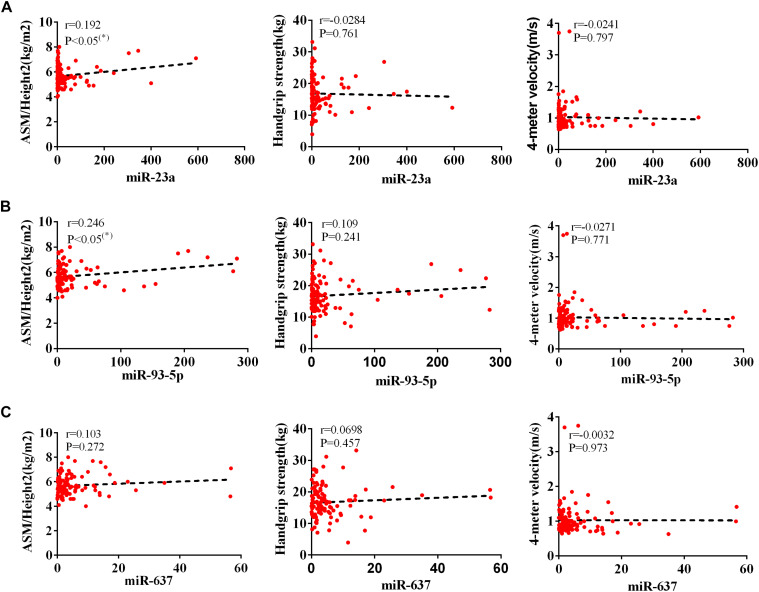
Correlation analysis between the changes in **(A)** miR-23a-3p, **(B)** miR-93-5p, and **(C)** miR-637 and ASM/height^2^ (kg/m^2^), handgrip strength (kg), and 4-m velocity (m/s) in women. The correlation analysis of miR-23a-3p and miR-93-5p and miR-637 with these indicators in female subjects. Pearson’s method was used for correlation analyses; *P < 0.05; *n* = 119.

**FIGURE 5:**
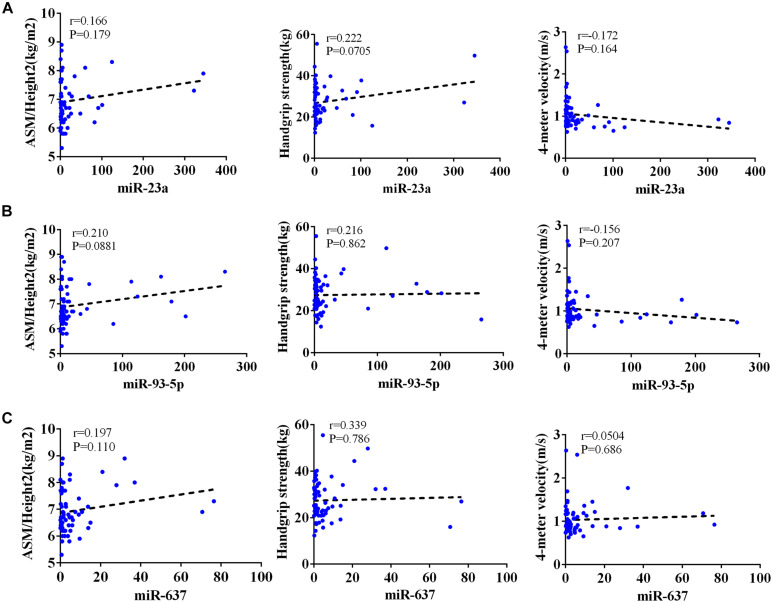
Correlation analysis between the changes in **(A)** miR-23a-3p, **(B)** miR-93-5p, and **(C)** miR-637 and ASM/height^2^ (kg/m^2^), handgrip strength (kg), and 4-m velocity (m/s) in men. The correlation analysis of miR-23a-3p and miR-93-5p and miR-637 with these indicators in male subjects. Pearson’s method was used for correlation analyses; *P < 0.05; *n* = 67.

The AWGS criteria used to diagnose muscular dystrophy are described below: low muscle mass, low muscle strength, and/or low physical strength. The detection was performed with ASM/height^2^, handgrip strength, and 4-m velocity, respectively, whose levels significantly decreased in the elderly with sarcopenia (Table 2). The expression levels of circulating miR-23a-3p, miR-93-5p, and miR-637 significantly decreased in elderly patients with sarcopenia. Then, we investigated whether there was any correlation between these miRNAs and the diagnostic indicators of sarcopenia (ASM/height^2^, handgrip strength, and 4-m speed). Our data indicated that the changes in miR-93-5p and miR-637 were remarkably related to ASM/height^2^ (*r* = 0.185, *p* < 0.05; *r* = 0.157, *p* < 0.05), while the changes in other miRNAs were not strongly correlated with these diagnostic indexes (Figure 3). To explore more the data presenting as cluster separation regarding the miRNA levels and the different sarcopenia predictors, we have analyzed the correlation between the lowest and highest quartiles of miRNA levels and the corresponding predictors of sarcopenia in each group, showing that the miR-93-5p expression was markedly allied to ASM/height^2^ (kg/m^2^), which is consistent with the results of previous analysis (Supplementary Figure 2). In further analyses with adjustments applied for sex, we found that the changes in miR-23a and miR-93-5p were significantly allied to ASM/height^2^ (*r* = 0.192, *p* < 0.05; *r* = 0.246, *p* < 0.05) in women (Figure 4). No significant correlations were found between miRNAs changes and these diagnostic indexes in men (Figure 5).

## Discussion

As a progressive and generalized skeletal muscle disorder involving the accelerated loss of muscle mass and function, sarcopenia is allied to aging, limiting the physical capabilities of the elderly (Marzetti et al., 2017; Larsson et al., 2019; Tournadre et al., 2019). Although the molecular mechanisms in sarcopenia remains entirely unclear, it is well documented that changes in miRNA during aging are major causes of sarcopenia (Siracusa et al., 2018; Jung et al., 2019; Yin et al., 2020). This study aims to investigate changes in specific circulating miRNAs associated with fracture risk in elderly patients with sarcopenia. From our data, it is noteworthy that the levels of plasma miR-23a-3p, miR-93-5p, and miR-637 significantly decreased in the sarcopenia group compared to those in the non-sarcopenia group, showing the association between the decrease in miR-23a-3p, miR-93-5p, and miR-637 with the predictor of sarcopenia, namely, the changes in miR-93-5p and miR-637 were significantly allied to ASM/height^2^. After adjusting sex, the changes in miR-23a and miR-93-5p were significantly allied to ASM/height^2^ in women, with no significant correlations between miRNAs changes and these diagnostic indexes in men. These results suggest the practicality and feasibility of detecting changes in miRNAs in the blood to detect early signs of sarcopenia and fractures during aging. Nevertheless, an accurate diagnosis of sarcopenia remains challenging. Thus, we urgently need to explore new biomarkers to predict sarcopenia in the elderly.

The circulating miRNA profile reflects the expression of miRNA in cells as a marker of biological processes occurring in cells. Because of their position in the hierarchy of gene expression before translating the genetic code into proteins, information obtained from their circulating levels may ultimately predict early biological responses to specific conditions, as fine tuners (Hamam et al., 2017; Valihrach et al., 2020). The discovery of relatively abundant levels of miRNAs in serum/plasma has opened the door to their exploitation as biomarkers for numerous diseases, including sarcopenia and aging-related diseases (Andersen and Tost, 2020; Müller et al., 2020; Sabre et al., 2020). Several diagnostic indicators of sarcopenia measured have been reported to effectively provide preventive measures in the elderly, including ASM/height^2^, handgrip strength, and 4-m velocity. It has been found that miRNAs exist in circulation in a consistent and reproducible manner, making them attractive for biomarker in patients with sarcopenia.

Selected miRNAs are involved in osteoblast formation and/or osteoclast formation and osteocyte function and/or clinical relevance, as they are allied to BMD and fracture risk. A growing evidence has suggested that several miRNAs expressed differentially in patients with osteoporotic and non-osteoporotic fractures (Mandourah et al., 2018; Zarecki et al., 2020). Specifically, miR-21, miR-23a, miR-24, miR-93, miR-100, miR-122a, miR-124a, miR-125b, and miR-148a were upregulated in osteoporotic serum, while miR-21, miR-23a, miR-24, miR-25, miR-100, and miR-125b were upregulated in osteoporotic bone tissues (Seeliger et al., 2014). Furthermore, miR-122a-5p, mir-125b-5p, and miR-21-5p were taken as osteoporotic markers for their fractures (Panach et al., 2015). Several circulating miRNAs have also been identified as closely related to sarcopenia in the elderly (Fan et al., 2016; Rusanova et al., 2018; Siracusa et al., 2018). Sarcopenia is closely related to osteoporosis, which together increases the risk of fractures (Atik, 2019; Greco et al., 2019). Thus, sarcopenia and osteoporosis are important risk factors of the frailty that leads to fracture. However, there is still no research to explore the common molecules leading to sarcopenia and osteoporosis in the elderly. Recently, our study has, for the first time, shown that miR-23a-3p, miR-93-5p, and miR-637, which are associated with fracture risk, are decreased in sarcopenia response in the elderly, while other miRNAs identified in this study were not altered, suggesting that sarcopenia and osteoporosis may be regulated by these miRNAs. Moreover, considering that most osteoblastogenesis and/or osteoclastogenesis-related miRNAs did not change in the current study, future studies are still needed to investigate the functions of miRNAs in patients with sarcopenia.

We did find that there were statistically significant differences in c-miRNA levels of sarcopenia and non-sarcopenia groups. Circulating miR-23a-3p, miR-93-5p, and miR-637 achieved significantly decreased values in the sarcopenia group. Thus, circulating miR-23a-3p, miR-93-5p, and miR-637 may act as a common signature for sarcopenia. Most of the previous studies indicated that sarcopenia is significantly associated with fractures (Edwards et al., 2015; Oliveira and Vaz, 2015; Yeung et al., 2019). Here, we report a significant decrease in plasma levels of fracture-related miR-23a-3p, miR-93-5p, and miR-637 in the elderly with sarcopenia, supporting its relation with sarcopenia and fractures. However, the mechanism of changes in the circulating levels of miRNA associated with fracture risk in sarcopenia remains unclear. Considering the critical function of miRNAs in regulating bone metabolism, we can reasonably assume that bone turnover could be evaluated by measuring changes in the level of bone-specific c-miRNAs that reflect bone resorption and bone formation. Besides, miRNAs can regulate many common signaling pathways in the metabolism of muscle and skeletal muscle cell (Horak et al., 2016; Zheng et al., 2018). Wnt/β-catenin signaling pathway regulates osteoblast activity, which is also associated with regeneration of muscles. Osteocytes can secrete osteosclerosis, Wnt3a, and prostaglandins, regulate Wnt/β-catenin pathway, and affect the metabolism and functions of bones and muscles, respectively. Muscle contraction is an important mechanical stimulus for bones, which can activate the Wnt/β-catenin pathway of bone cells by enhancing the sheer force of fluid flow, thereby regulating osteoblast activity (Wannenes et al., 2014; Huang et al., 2017). Another signaling pathway closely related to muscle and bone is the PI3K/Akt pathway, through which insulin-like growth factor-1 exerts a beneficial effect on muscle and bone to promote anabolic metabolism (Yang et al., 2010; Cui et al., 2020). Finally, given that muscles and bones are regulated by many common genes, endocrine regulatory networks, and signaling pathways, both osteoporosis and sarcopenia are key factors to fractures. Including sarcopenia status may be useful in the fracture risk model. In the present study, our results on plasma circulating miRNA expression provide new insights in diagnosing and treating sarcopenia.

Sarcopenia can be diagnosed as low muscle mass, low muscle strength, and/or low physical performance by AWGS criteria (Chen et al., 2014). Currently, the diagnostic criteria for sarcopenia are ASM/height^2^, handgrip strength, and 4-m velocity, which can indicate the severity of sarcopenia. Interestingly, we found that the relative expressions of miR-93-5p and miR-637 were significantly positively associated with ASM/height^2^, respectively, with a significant relation between the changes in miR-23a and miR-93-5p and ASM/height^2^ in women while no significant correlations in men after adjusting sex. For one thing, researchers have consistently found that circulating miR-23a, miR-93-5p, and miR-637 are associated with fractures in patients with osteoporosis (Sansoni et al., 2018). Frequently, sarcopenia and osteoporosis are major syndromes in elderly patients, which are associated with weakness, including weight loss, low muscle strength, slow walking speed and instability, and more importantly, often leading to falls and fractures. Most studies have suggested that the incidence of sarcopenia differs among older men and women after a fragility fracture (Anderson et al., 2017; Laurent et al., 2019; Wong et al., 2019). Sarcopenia has some risk factors, and the decline in sex hormones may be one reason for sex differences, which may explain the difference in the correlation between changes in miRNAs levels and the diagnostic indicators of sarcopenia in different genders in our study. In addition, 63.4% of the subjects were female, and only 36.6% were male in our study. The possibility of the lack of this correlation in the male may be due to fewer participants.

There are still some limitations in our research. First, it is a single-center clinical trial with a small sample, the results of which need to be verified in a large sample, coverage area, and multicenter. If more patients of different races could be included, the results would be more reliable. Second, only eight targeted miRNAs that were associated with a fracture in the literature were examined because of cost; thus, there may be other unstudied miRNAs that may be better indicators of osteoporosis and sarcopenia status. Moreover, while the people in the study were relatively healthy, there may still be some patients with certain diseases. miRNA expression may alter with disease conditions, such as degenerative diseases, malignancies, and autoimmune diseases.

In summary, it has been proven that c-miRNAs may be considered as possible biomarkers for sarcopenia as a new diagnostic tool to monitor response to treatment. Further research into sarcopenia and osteoporosis-related fractures is also urgently needed to understand their relationships and mechanisms. All these mentioned above can provide more evidence to develop potential interventions and improve clinical outcomes.

## Data Availability Statement

The original contributions presented in the study are included in the article/Supplementary Material, further inquiries can be directed to the corresponding author/s.

## Ethics Statement

The studies involving human participants were reviewed and approved by The Ethics Committee of Ningbo No. 2 Hospital. The patients/participants provided their written informed consent to participate in this study. Written informed consent was obtained from the individual(s) for the publication of any potentially identifiable images or data included in this article.

## Author Contributions

HY was in charge of conceiving and designing and revising the manuscript. NH was in charge of performing the experiments and writing the manuscript. NH, YLZ, and ZZ were in charge of collecting and analyzing the data. NH, BF, DW, and SZ were in charge of contributing the reagents, materials, and analysis tools used in this study. All authors contributed to the article and approved the submitted version.

## Conflict of Interest

The authors declare that the research was conducted in the absence of any commercial or financial relationships that could be construed as a potential conflict of interest.

## Supplementary Material

Supplementary Figure 1 Fracture risk-associated circulating miR-23a-3p, miR-93-5p and miR-637 decrease in response to sarcopenia without diabetes in the elderly. The expression level of miRNAs were normalized using spike-in cel-miR-39; unpaired *T*-test was used for these data; *compared to non-sarcopenia of the elderly; **P* < 0.05; *n* = 40.

Supplementary Figure 2 Correlation analysis between the lowest and highest quartile of the miR-23a-3p **(A)**, miR-93-5p **(B)**, miR-637 **(C)**, and ASM/Height^2^ (kg/m^2^), Handgrip strength (kg) and 4-m velocity (m/s). Pearson’s method was used for Correlation analyses; **P* < 0.05*; n* = 92.

## References

[B1] Andersen G. B. Tost J. (2020). Circulating miRNAs as biomarker in cancer. *Recent Results Cancer Res.* 215 277–298. 10.1007/978-3-030-26439-0_15 31605235

[B2] Anderson L. J. Liu H. Garcia J. M. (2017). Sex differences in muscle wasting. *Adv. Exp. Med. Biol.* 1043 153–197. 10.1007/978-3-319-70178-3_9 29224095

[B3] Angulo J. C. Assar M. E. Rodriguezmanas L. (2016). Frailty and sarcopenia as the basis for the phenotypic manifestation of chronic diseases in older adults. *Mol. Aspects Med.* 50 1–32. 27370407 10.1016/j.mam.2016.06.001

[B4] Anton S. D. Hida A. Robert M. Andrew L. Solberg L. M. Mainous A. G. (2018). Nutrition and Exercise in Sarcopenia. *Curr. Protein Peptide Sci.* 19 649–667. 28029078 10.2174/1389203717666161227144349

[B5] Atik O. (2019). There is an association between Sarcopenia, osteoporosis, and the risk of hip fracture. *Eklem Hastalik Cerrahisi* 30:1. 10.5606/ehc.2019.001 30885101

[B6] Bauer J. M. Verlaan S. Bautmans I. Brandt K. Donini L. M. Maggio M. (2015). Effects of a vitamin D and leucine-enriched whey protein nutritional supplement on measures of sarcopenia in older adults, the PROVIDE study: a randomized, double-blind, placebo-controlled trial. *J. Am. Med. Dir. Assoc.* 16 740–747. 10.1016/j.jamda.2015.05.021 26170041

[B7] Bonewald L. F. Kiel D. P. Clemens T. L. Esser K. Orwoll E. S. O’Keefe R. J. (2013). Forum on bone and skeletal muscle interactions: summary of the proceedings of an ASBMR workshop. *J. Bone Miner. Res.* 28 1857–1865. 10.1002/jbmr.1980 23671010 PMC3749267

[B8] Chen L. K. Liu L. K. Woo J. Assantachai P. Auyeung T. W. Bahyah K. S. (2014). Sarcopenia in Asia: consensus report of the Asian Working Group for Sarcopenia. *J. Am. Med. Dir. Assoc.* 15 95–101. 10.1016/j.jamda.2013.11.025 24461239

[B9] Chen Z. Bemben M. G. Bemben D. A. (2019). Bone and muscle specific circulating microRNAs in postmenopausal women based on osteoporosis and sarcopenia status. *Bone* 120 271–278. 10.1016/j.bone.2018.11.001 30408612

[B10] Cui C. Han S. Shen X. He H. Chen Y. Zhao J. (2020). ISLR regulates skeletal muscle atrophy via IGF1-PI3K/Akt-Foxo signaling pathway. *Cell Tissue Res.* 381 479–492. 10.1007/s00441-020-03251-4 32696215

[B11] Ding W. Ding S. Li J. Peng Z. Hu P. Zhang T. (2019). Aberrant expression of miR-100 in plasma of patients with osteoporosis and its potential diagnostic value. *Clin. Lab.* 65 9. 10.7754/Clin.Lab.2019.190327 31532098

[B12] Edwards M. H. Dennison E. M. Aihie Sayer A. Fielding R. Cooper C. (2015). Osteoporosis and sarcopenia in older age. *Bone* 80 126–130. 10.1016/j.bone.2015.04.016 25886902 PMC4601530

[B13] Eshaghi S. Morteza T. Khadijeh I. Knechtle B. Nikolaidis P. T. Chtourou H. (2020). The effect of aerobic training and vitamin D supplements on the neurocognitive functions of elderly women with sleep disorders. *Biol. Rhythm Res.* 51 1–8.

[B14] Fan J. Kou X. Yang Y. Chen N. (2016). MicroRNA-regulated proinflammatory cytokines in Sarcopenia. *Mediators Inflamm.* 2016:1438686. 10.1155/2016/1438686 27382188 PMC4921629

[B15] Gámez B. Rodriguez-Carballo E. Ventura F. (2014). MicroRNAs and post-transcriptional regulation of skeletal development. *J. Mol. Endocrinol.* 52 R179–R197. 10.1530/jme-13-0294 24523514

[B16] Gareev I. Beylerli O. Yang G. Sun J. Pavlov V. Izmailov A. (2020). The current state of MiRNAs as biomarkers and therapeutic tools. *Clin. Exp. Med.* 20 349–359. 10.1007/s10238-020-00627-2 32399814

[B17] Girgis C. M. (2015). Integrated therapies for osteoporosis and sarcopenia: from signaling pathways to clinical trials. *Calcif. Tissue Int.* 96 243–255. 10.1007/s00223-015-9956-x 25633430

[B18] Greco E. A. Pietschmann P. Migliaccio S. (2019). Osteoporosis and Sarcopenia increase frailty syndrome in the elderly. *Front. Endocrinol. (Lausanne)* 10:255. 10.3389/fendo.2019.00255 31068903 PMC6491670

[B19] Hamam R. Hamam D. Alsaleh K. A. Kassem M. Zaher W. Alfayez M. (2017). Circulating microRNAs in breast cancer: novel diagnostic and prognostic biomarkers. *Cell Death Dis.* 8:e3045. 10.1038/cddis.2017.440 28880270 PMC5636984

[B20] Hong I. S. Lee H. Y. Choi S. W. Kim H. S. Yu K. R. Seo Y. (2013). The effects of hedgehog on RNA binding protein Msi1 during the osteogenic differentiation of human cord blood-derived mesenchymal stem cells. *Bone* 56 416–425. 10.1016/j.bone.2013.07.016 23880227

[B21] Horak M. Novak J. Bienertova-Vasku J. (2016). Muscle-specific microRNAs in skeletal muscle development. *Dev. Biol.* 410 1–13. 10.1016/j.ydbio.2015.12.013 26708096

[B22] Huang J. Romero-Suarez S. Lara N. Mo C. Kaja S. Brotto L. (2017). Crosstalk between MLO-Y4 osteocytes and C2C12 muscle cells is mediated by the Wnt/β-catenin pathway. *JBMR Plus* 1 86–100. 10.1002/jbm4.10015 29104955 PMC5667655

[B23] Hunter G. R. Wetzstein C. J. Fields D. A. Brown A. Bamman M. M. (2000). Resistance training increases total energy expenditure and free-living physical activity in older adults. *J. Appl. Physiol. (1985)* 89 977–984. 10.1152/jappl.2000.89.3.977 10956341

[B24] Irandoust K. Taheri M. Shavikloo J. (2018). The effect of water-based aerobic training on the dynamic balance and walking speed of obese elderly men with low back pain. *Sleep Hypnosis* 20 233–240.

[B25] Irandoust K. Taheri M. Mirmoezzi M. H’mida C. Chtourou H. Trabelsi K. (2019). The effect of aquatic exercise on postural mobility of healthy older adults with endomorphic somatotype. *Int. J. Environ. Res. Public Health* 16:4387. 10.3390/ijerph16224387 PMC6888232 31717625

[B26] Jung H. J. Lee K. P. Kwon K. S. Suh Y. (2019). MicroRNAs in skeletal muscle aging: current issues and perspectives. *J. Gerontol. A Biol. Sci. Med. Sci.* 74 1008–1014. 10.1093/gerona/gly207 30215687 PMC6580686

[B27] Kelch S. Balmayor E. R. Seeliger C. Vester H. Kirschke J. S. van Griensven M. (2017). miRNAs in bone tissue correlate to bone mineral density and circulating miRNAs are gender independent in osteoporotic patients. *Sci. Rep.* 7:15861. 10.1038/s41598-017-16113-x 29158518 PMC5696459

[B28] Kocijan R. Muschitz C. Geiger E. Skalicky S. Baierl A. Dormann R. (2016). Circulating microRNA signatures in patients with idiopathic and postmenopausal osteoporosis and fragility fractures. *J. Clin. Endocrinol. Metab.* 101 4125–4134. 10.1210/jc.2016-2365 27552543

[B29] Kosaka N. Iguchi H. Ochiya T. (2010). Circulating microRNA in body fluid: a new potential biomarker for cancer diagnosis and prognosis. *Cancer Sci.* 101 2087–2092. 10.1111/j.1349-7006.2010.01650.x 20624164 PMC11159200

[B30] Landi F. Calvani R. Cesari M. Tosato M. Martone A. M. Ortolani E. (2018). Sarcopenia: an overview on current definitions, diagnosis and treatment. *Curr. Protein Pept. Sci.* 19 633–638. 10.2174/1389203718666170607113459 28595526

[B31] Larsson L. Degens H. Li M. Salviati L. Lee Y. Thompson W. J. (2019). Sarcopenia: aging-related loss of muscle mass and function. *Physiol. Rev.* 99 427–511. 30427277 10.1152/physrev.00061.2017 PMC6442923

[B32] Laurent M. R. Dedeyne L. Dupont J. Mellaerts B. Dejaeger M. Gielen E. (2019). Age-related bone loss and sarcopenia in men. *Maturitas* 122 51–56. 10.1016/j.maturitas.2019.01.006 30797530

[B33] Lisse T. S. Chun R. F. Rieger S. Adams J. S. Hewison M. (2013). Vitamin D activation of functionally distinct regulatory miRNAs in primary human osteoblasts. *J. Bone Miner. Res.* 28 1478–1488. 10.1002/jbmr.1882 23362149 PMC3663893

[B34] Mandourah A. Y. Ranganath L. Barraclough R. Vinjamuri S. Hof R. V. Hamill S. (2018). Circulating microRNAs as potential diagnostic biomarkers for osteoporosis. *Sci. Rep.* 8:8421. 10.1038/s41598-018-26525-y 29849050 PMC5976644

[B35] Marques A. Queirós C. (2018). “Frailty, Sarcopenia and falls,” in *Fragility Fracture Nursing: Holistic Care and Management of the Orthogeriatric Patient*, eds Hertz K. Santy-Tomlinson J. (Cham: Springer), 15–26. 31314236

[B36] Marzetti E. Calvani R. Tosato M. Cesari M. Bari M. D. Cherubini A. (2017). Sarcopenia: an overview. *Aging Clin. Exp. Res.* 29 11–17. 28155183 10.1007/s40520-016-0704-5

[B37] Müller S. Janke F. Dietz S. Sültmann H. (2020). Circulating MicroRNAs as potential biomarkers for lung cancer. *Recent Results Cancer Res.* 215 299–318. 10.1007/978-3-030-26439-0_16 31605236

[B38] Oliveira A. Vaz C. (2015). The role of sarcopenia in the risk of osteoporotic hip fracture. *Clin. Rheumatol.* 34 1673–1680. 10.1007/s10067-015-2943-9 25912213

[B39] Panach L. Mifsut D. Tarín J. J. Cano A. García-Pérez M. (2015). Serum circulating MicroRNAs as biomarkers of osteoporotic fracture. *Calcif. Tissue Int.* 97 495–505. 10.1007/s00223-015-0036-z 26163235

[B40] Perksanusak T. Panyakhamlerd K. Hirankarn N. Suwan A. Vasuratna A. Taechakraichana N. (2018). Correlation of plasma microRNA-21 expression and bone turnover markers in postmenopausal women. *Climacteric* 21 581–585. 10.1080/13697137.2018.1507020 30232913

[B41] Qadir A. S. Um S. Lee H. Baek K. Seo B. M. Lee G. (2015). miR-124 negatively regulates osteogenic differentiation and in vivo bone formation of mesenchymal stem cells. *J. Cell. Biochem.* 116 730–742. 10.1002/jcb.25026 25424317

[B42] Reiss J. Iglseder B. Alzner R. Mayr-Pirker B. Pirich C. Kässmann H. (2019). Sarcopenia and osteoporosis are interrelated in geriatric inpatients. *Z. Gerontol. Geriatr.* 52 688–693. 10.1007/s00391-019-01553-z 31049683 PMC6817738

[B43] Rusanova I. Diaz-Casado M. E. Fernández-Ortiz M. Aranda-Martínez P. Guerra-Librero A. García-García F. J. (2018). Analysis of plasma MicroRNAs as predictors and biomarkers of aging and frailty in humans. *Oxid. Med. Cell. Longev.* 2018:7671850. 10.1155/2018/7671850 30116492 PMC6079380

[B44] Sabre L. Punga T. Punga A. R. (2020). Circulating miRNAs as potential biomarkers in myasthenia gravis: tools for personalized medicine. *Front. Immunol.* 11:213. 10.3389/fimmu.2020.00213 32194544 PMC7065262

[B45] Saliminejad K. Khorram Khorshid H. R. Soleymani Fard S. Ghaffari S. H. (2019). An overview of microRNAs: biology, functions, therapeutics, and analysis methods. *J. Cell. Physiol.* 234 5451–5465. 10.1002/jcp.27486 30471116

[B46] Sansoni V. Perego S. Vernillo G. Barbuti A. Merati G. La Torre A. (2018). Effects of repeated sprints training on fracture risk-associated miRNA. *Oncotarget* 9 18029–18040. 10.18632/oncotarget.24707 29719588 PMC5915055

[B47] Seeliger C. Karpinski K. Haug A. T. Vester H. Schmitt A. Bauer J. S. (2014). Five freely circulating miRNAs and bone tissue miRNAs are associated with osteoporotic fractures. *J. Bone Miner. Res.* 29 1718–1728. 10.1002/jbmr.2175 24431276

[B48] Seghatoleslami A. Afif A. H. Irandoust K. Taheri M. (2018). The impact of pilates exercises on motor control of inactive middle-aged women. *Sleep Hypnosis* 20 262–266.

[B49] Siracusa J. Koulmann N. Banzet S. (2018). Circulating myomiRs: a new class of biomarkers to monitor skeletal muscle in physiology and medicine. *J. Cachexia Sarcopenia Muscle* 9 20–27. 10.1002/jcsm.12227 29193905 PMC5803618

[B50] Soriano-Arroquia A. House L. Tregilgas L. Canty-Laird E. Goljanek-Whysall K. (2016). The functional consequences of age-related changes in microRNA expression in skeletal muscle. *Biogerontology* 17 641–654. 10.1007/s10522-016-9638-8 26922183 PMC4889642

[B51] Steihaug O. M. Gjesdal C. G. Bogen B. Kristoffersen M. H. Lien G. Ranhoff A. H. (2017). Sarcopenia in patients with hip fracture: a multicenter cross-sectional study. *PLoS One* 12:e0184780. 10.1371/journal.pone.0184780 28902873 PMC5597226

[B52] Tang L. Yin Y. Liu J. Li Z. Lu X. (2017). MiR-124 attenuates osteoclastogenic differentiation of bone marrow monocytes via targeting Rab27a. *Cell. Physiol. Biochem.* 43 1663–1672. 10.1159/000484027 29045940

[B53] Tarantino U. Baldi J. Scimeca M. Piccirilli E. Piccioli A. Bonanno E. (2016). The role of sarcopenia with and without fracture. *Injury* 47(Suppl. 4) S3–S10. 10.1016/j.injury.2016.07.057 27496721

[B54] Tournadre A. Vial G. Capel F. Soubrier M. Boirie Y. (2019). Sarcopenia. *Joint Bone Spine* 86 309–314. 10.1016/j.jbspin.2018.08.001 30098424

[B55] Valihrach L. Androvic P. Kubista M. (2020). Circulating miRNA analysis for cancer diagnostics and therapy. *Mol. Aspects Med.* 72:100825. 10.1016/j.mam.2019.10.002 31635843

[B56] Van Roie E. Delecluse C. Coudyzer W. Boonen S. Bautmans I. (2013). Strength training at high versus low external resistance in older adults: effects on muscle volume, muscle strength, and force-velocity characteristics. *Exp. Gerontol.* 48 1351–1361. 10.1016/j.exger.2013.08.010 23999311

[B57] Verdelli C. Sansoni V. Perego S. Favero V. Vitale J. Terrasi A. (2020). Circulating fractures-related microRNAs distinguish primary hyperparathyroidism-related from estrogen withdrawal-related osteoporosis in postmenopausal osteoporotic women: a pilot study. *Bone* 137:115350. 10.1016/j.bone.2020.115350 32380256

[B58] Wannenes F. Papa V. Greco E. A. Fornari R. Marocco C. Baldari C. (2014). Abdominal fat and Sarcopenia in women significantly alter osteoblasts homeostasis in vitro by a WNT/ β -catenin dependent mechanism. *Int. J. Endocrinol.* 2014:278316. 10.1155/2014/278316 24963291 PMC4054618

[B59] Westcott W. L. (2009). Strength training for frail older adults. *J. Active Aging* 8 52–59.

[B60] Wong R. M. Y. Wong H. Zhang N. Chow S. K. H. Chau W. W. Wang J. (2019). The relationship between sarcopenia and fragility fracture-a systematic review. *Osteoporos. Int.* 30 541–553. 10.1007/s00198-018-04828-0 30610245

[B61] Yamada M. Kimura Y. Ishiyama D. Nishio N. Otobe Y. Tanaka T. (2019). Synergistic effect of bodyweight resistance exercise and protein supplementation on skeletal muscle in sarcopenic or dynapenic older adults. *Geriatr. Gerontol. Int.* 19 429–437. 30864254 10.1111/ggi.13643

[B62] Yang M. Liu Y. Zuo Y. Tang H. (2019). Sarcopenia for predicting falls and hospitalization in community-dwelling older adults: EWGSOP versus EWGSOP2. *Sci. Rep.* 9:17636. 10.1038/s41598-019-53522-6 31776354 PMC6881315

[B63] Yang S. Y. Hoy M. Fuller B. Sales K. M. Seifalian A. M. Winslet M. C. (2010). Pretreatment with insulin-like growth factor I protects skeletal muscle cells against oxidative damage via PI3K/Akt and ERK1/2 MAPK pathways. *Lab. Invest.* 90 391–401. 10.1038/labinvest.2009.139 20084055

[B64] Yeung S. S. Y. Reijnierse E. M. Pham V. K. Trappenburg M. C. Lim W. K. Meskers C. G. M. (2019). Sarcopenia and its association with falls and fractures in older adults: a systematic review and meta-analysis. *J. Cachexia Sarcopenia Muscle* 10 485–500. 10.1002/jcsm.12411 30993881 PMC6596401

[B65] Yin J. Qian Z. Chen Y. Li Y. Zhou X. (2020). MicroRNA regulatory networks in the pathogenesis of sarcopenia. *J. Cell. Mol. Med.* 24 4900–4912. 10.1111/jcmm.15197 32281300 PMC7205827

[B66] Zarecki P. Hackl M. Grillari J. Debono M. Eastell R. (2020). Serum microRNAs as novel biomarkers for osteoporotic vertebral fractures. *Bone* 130:115105. 10.1016/j.bone.2019.115105 31669252

[B67] Zendjabil M. Favard S. Tse C. Abbou O. Hainque B. (2017). [The microRNAs as biomarkers: what prospects?]. *C. R. Biol.* 340 114–131. 10.1016/j.crvi.2016.12.001 28081967

[B68] Zhang J. F. Fu W. M. He M. L. Wang H. Wang W. M. Yu S. C. (2011). MiR-637 maintains the balance between adipocytes and osteoblasts by directly targeting Osterix. *Mol. Biol. Cell* 22 3955–3961. 10.1091/mbc.E11-04-0356 21880893 PMC3204058

[B69] ZhangL.GuoQ.FengB.-L.WangC.-Y.HanP.-P.HuJ. (2019). A cross-sectional study of the association between arterial stiffness and Sarcopenia in Chinese community-dwelling elderly using the Asian working group for sarcopenia criteria. *J. Nutr. Health Aging* 23 195–201.3069763010.1007/s12603-018-1147-9

[B70] Zheng Y. Kong J. Li Q. Wang Y. Li J. (2018). Role of miRNAs in skeletal muscle aging. *Clin. Interv Aging* 13 2407–2419. 10.2147/cia.s169202 30538437 PMC6254589

[B71] Zou L. Zhang G. Liu L. Chen C. Cao X. Cai J. (2017). A MicroRNA-124 polymorphism is associated with fracture healing via modulating BMP6 expression. *Cell. Physiol. Biochem.* 41 2161–2170. 10.1159/000475570 28441666

